# A high-quality, long-read genome assembly of the whitelined sphinx moth (Lepidoptera: Sphingidae: *Hyles lineata*) shows highly conserved melanin synthesis pathway genes

**DOI:** 10.1093/g3journal/jkad090

**Published:** 2023-04-29

**Authors:** R Keating Godfrey, Sarah E Britton, Shova Mishra, Jay K Goldberg, Akito Y Kawahara

**Affiliations:** McGuire Center for Lepidoptera and Biodiversity, Florida Museum of Natural History, University of Florida, 3215 Hull Rd, Gainesville, FL 32611, USA; Department of Ecology and Evolutionary Biology, University of Arizona, 1041 E. Lowell St, Tucson, AZ 85721, USA; Department of Entomology and Nematology, University of Florida, 1881 Natural Area Dr., Gainesville, FL 32608, USA; Department of Ecology and Evolutionary Biology, University of Arizona, 1041 E. Lowell St, Tucson, AZ 85721, USA; McGuire Center for Lepidoptera and Biodiversity, Florida Museum of Natural History, University of Florida, 3215 Hull Rd, Gainesville, FL 32611, USA

**Keywords:** Lepidoptera, whitelined sphinx, *Hyles lineata*, genome assembly, melanin synthesis genes

## Abstract

The sphinx moth genus *Hyles* comprises 29 described species inhabiting all continents except Antarctica. The genus diverged relatively recently (40–25 MYA), arising in the Americas and rapidly establishing a cosmopolitan distribution. The whitelined sphinx moth, *Hyles lineata*, represents the oldest extant lineage of this group and is one of the most widespread and abundant sphinx moths in North America. *Hyles lineata* exhibits the large body size and adept flight control characteristic of the sphinx moth family (Sphingidae), but it is unique in displaying extreme larval color variation and broad host plant use. These traits, in combination with its broad distribution and high relative abundance within its range, have made *H. lineata* a model organism for studying phenotypic plasticity, plant–herbivore interactions, physiological ecology, and flight control. Despite being one of the most well-studied sphinx moths, little data exist on genetic variation or regulation of gene expression. Here, we report a high-quality genome showing high contiguity (N50 of 14.2 Mb) and completeness (98.2% of Lepidoptera BUSCO genes), an important first characterization to facilitate such studies. We also annotate the core melanin synthesis pathway genes and confirm that they have high sequence conservation with other moths and are most similar to those of another, well-characterized sphinx moth, the tobacco hornworm (*Manduca sexta*).

## Introduction

The whitelined sphinx, *Hyles lineata* (Sphingidae), was first described by zoologist Johan Christian Fabricius in 1775 from a pinned museum specimen. At that time, this conspicuous, abundant moth was already well-known to the Indigenous Peoples of North America, appearing on pottery and cave paintings, and in cuisine (Nabhan *et al*. 1989; VanPool 2009). Indeed, the moth-like figure depicted hovering around the flower of the nightshade, sacred datura (*Datura wrightii*) in Chumash rock art in southern California is thought to represent *H. lineata* (Robinson *et al*. 2020). But, while most sphinx moths are nocturnal and nectar from cacti or nightshades (Heinrich 1993; Tuttle 2007), *H*. *lineata* can also be observed nectaring during the day from a variety of plants (Raguso *et al*. 1996; Tuttle 2007; Alarcón *et al*. 2008). Additionally, *H. lineata* often occurs in high abundance, with multiple adults nectaring from the same plant or dozens of prepupal caterpillars migrating in gregarious mobs. Caterpillars can be so abundant that the Tohono O’odham of the southwest used them as a food source called *makkum* (Fontana 1974; Crosswhite 1981). These characteristics make *H. lineata* unique among sphinx moths. The species is particularly interesting to studies of trait evolution because modern phylogenetic analyses provide strong, consistent support for *H. lineata* as sister to all other *Hyles* (Hundsdoerfer *et al*. 2009).

In addition to unique behaviors and life history traits, *H. lineata* exhibit highly variable caterpillar color polymorphisms and are used as a scientific model of phenotypic plasticity. Pigmentation in the cuticle and wings of insects is largely determined by the highly conserved melanogenesis metabolic pathway, including “core” melanin synthesis genes that encode the necessary enzymes (Sugumaran and Barek 2016). However, while cuticular color and pattern are, in part, inherited through allelic variation, the degree of melanin pigmentation is determined by environmental variables including temperature, photoperiod, and crowding (Francois and Davidowtiz 2020; Britton and Davidowitz in review). Purifying selection drives gene sequence conservation and phenotype variation is driven largely by gene regulation, rather than allelic differences (Kuwalekar *et al*. 2020). The pleiotropic nature of these core genes likely limits evolution in the protein-coding sequences, as well, especially since the melanin pathway is important for other essential processes including sclerotization and immune response (Wittkopp and Beldade 2009). Genetic networks underlying adult wing patterns and variation in caterpillar cuticular color are being teased apart for several Lepidoptera (Futahashi *et al*. 2010; Yamaguchi *et al*. 2013; Matsuoka and Monteiro 2018; Kuwalekar *et al*. 2020) and the availability of sequenced genomes will facilitate a mechanistic study of gene function and regulation.

Here, we present a high-quality long-read genome assembly for *H. lineata* and curate the annotation of several genes in the melanin synthesis pathway. Our assembly shows high contiguity (N50 = 14.2 Mb) and completeness (98.2% of Lepidoptera BUSCO genes recovered). Additionally, predicted protein sequences of the core melanin synthesis pathway are highly conserved and show the greatest amount of similarity to the related tobacco hornworm, *Manduca sexta* (Lepidoptera: Sphingidae).

## Methods

### DNA isolation and sequencing

The specimen used for whole-genome sequencing originated from a lab colony maintained at the University of Arizona. The entire specimen was consumed in the process of isolating high-molecular-weight DNA; therefore, a sibling of the same generation was vouchered at the Florida Museum of Natural History's McGuire Center for Lepidoptera and Biodiversity (MGCL_LEP-89333). A male puparium was stored in 100% ethanol at −20°C for 2 weeks prior to DNA isolation. DNA was isolated from thoracic tissue using the Qiagen DNeasy Blood and Tissue Kit (Cat. # 69504) adapted for extraction of high-molecular-weight DNA.

SMRT bell libraries were prepared at the University of Florida Interdisciplinary Center for Biotechnology Research (ICBR; RRID:SCR_019152) and sequenced on the PacBio SEQUEL IIe according to the recommended protocol (P/N 101-853-100 v. 05, August 2021) with a few modifications. This procedure resulted in ∼650 ng of SMRTbell library fragments of ∼11.5 kb size. The on-plate loading concentration was 75 pM. The instrument used PacBio Sequencing Kit 2.0 (Cat. # 101-389-001) and Instrument Control SW Version 11.0 (SMRT Link 11.0). All other steps for sequencing were done according to the recommended protocol by using the PacBio sequencing calculator. One SEQUEL IIe SMRT cell with a 30 h movie resulted in ∼6.5 million polymerase reads (∼550 Gb total output) and ∼3.5 million Hi-Fi reads (∼27 Gb) with an average polymerase read length of 84.7 kb and the longest subread N50 of ∼11.8 kb (see Supplemental Materials for detailed information on rearing, DNA isolation, and sequencing).

### Genome size

Genome size, heterozygosity, and repetitiveness were first estimated from the consensus reads using a k-mer distribution approach. K-mer counter v.3.2.1 (RRID: SCR_001245) was used with a *k-mer* length of 29 (-m 29) to generate a histogram of *k-mer* frequencies using the transform function. We visualized the *k-mer* count histogram and assessed *k-mer* profiles using the browser-based GenomeScope 2.0 (RRID:SCR_017014) with *k-mer* length set to 29 and ploidy equal to 2 (Supplementary Fig. 1).

### Assembly and analysis

Reads were assembled into contigs using Hifiasm v.0.16.1 r307 (RRID:SCR_021069) with aggressive duplicate purging (option -l 3) and the resulting assembly graph of primary contigs (*.p_ctg.gfa) was used for all downstream analyses. Contiguity and completeness of the assembly were assessed using assembly_stats.py (Manchanda *et al*. 2020) and BUSCO (Benchmarking for University Single Copy Orthologs) v.5.2.0 with 5,289 single-copy orthologous genes in the lepidoptera_odb10 data set (RRID: SCR_015008; Manni *et al*. 2021). Despite our use of the most aggressive duplicate purging setting in Hifiasm, this primary assembly showed a higher percentage of complete, duplicated BUSCO matches than expected for an insect genome (3.0%, Table 1), indicating that not all homologous haplotigs were collapsed during assembly. Therefore, we employed the Purge Haplotigs pipeline, purge_haplotigs v.1.1.2 (Roach *et al*. 2018). Raw reads were first mapped to the primary assembly using minimap v.2.21 (RRID:SCR_018550; Li 2018) to create a coverage histogram for duplicate purging. Visual inspection of this histogram was used to choose low and high read depth cutoff values along with a midpoint value that fell between diploid peaks. Contigs were assigned as suspected haplotigs if the 80% of the contig showed diploid-level coverage (-s 80) and as junk if coverage was 80% above or below the read depth cut offs (-j 80). This resulted in a 3.9% reduction in assembly size but a lower percentage of complete, duplicated BUSCO hits (Initial assembly vs Curated assembly, Table 1). Contamination of this purged assembly was assessed using BlobTools v1.0 (Laetsch and Blaxter 2017) and was determined to be insignificant, with 3 low-coverage *Firmicutes* hits (Supplementary Fig. 2), a bacteria phylum common in insect gut microbiota (Yun *et al*. 2014; for contig identities from BlobTools, see blobtools_table.txt file available on DRYAD: https://doi.org/10.5061/dryad.95x69p8q0).

**Table 1.** Assembly statistics and comparison with select Sphingidae.

Assembly	*H. lineata*		*H. euphorbiae^a^*	*H. vespertilio^a^*	*M. sexta^b^*
	Curated contigs FLMNH_Hlin_1.0	Initial assembly	ilHylEuph1_pri	myriam-sen2290-mb-hirise-a4l1o	JHU_Msex_v1.0
Assembly length	452,616,994	471,105,525	504,310,614	651,427,907	468,966,500
Contig N50	14,292,520	14,181,941	2,758,341	7,263,332	402,416
Contigs	52	238	537	322	6,517*^c^*
Repeat content	31.16	38.16	47.1	53.39	33.91
BUSCO (*N* = 5,286)					
Complete	98.2	98.9	98.3	98.3	98.1
Single copy	97.6	95.9	97.9	95.4	93.1
Duplicated	0.6	3	0.3	2.9	5
Fragmented	0.4	0.2	0.5	0.7	0.7
Missing	1.4	0.9	1.3	1	1.2

a Table 2 from Hundsdoerfer *et al*. (preprint). *^b^*Supplementary Table 2. *^c^*Table 2 from Gershman *et al*. (2021).

### RNA-seq

We extracted RNA from specific tissues of a single late instar larva (head capsule, midgut) and 2 adult individuals of each sex (antennae, legs, female distal abdominal segments/ovipositor) separately. Concentration was assessed via Nanodrop and then the tissue-specific extractions were pooled such that each tissue's RNA concentration was roughly equal in the sequenced samples (larval, adult male, adult female). These tissues/stages were selected to maximize the gene content represented in our final annotation. All specimens were obtained from the same source colony as the individual used for gDNA extraction/sequencing. RNA was isolated using the ZYMO (Irvine, CA, USA) direct-zol miniprep kit (Cat. # R2050) and sequenced using NovaSeq (Illumina, San Diego, CA, USA) paired-end (150 bp) sequencing performed by Novogene (Sacramento, CA, USA).

### Structural annotation and gene prediction

Structural annotation was carried out with BRAKER v2.1.5 (RRID:SCR_018964; Hoff *et al*. 2019), which relies on BamTools (RRID:SCR_015987; Barnett *et al*. 2011), GeneMark-EP+ (RRID:SCR_011930; Bruna *et al*. 2020), DIAMOND (RRID:SCR_016071; Buchfink *et al*. 2015), and Augustus (RRID:SCR_008417; Stanke *et al*. 2008). The Curated assembly (Table 1) was first soft masked using a repeat library produced by RepeatModeler v2.0. and RepeatMasker v4.1.1. Raw RNA-seq fastq files were cleaned and trimmed using Trimmomatic (RRID:SCR_011848; Bolger *et al*. 2014), then mapped to the soft-masked reference assembly using HISAT2 (RRID:SCR_015530; Kim *et al*. 2019) and sorted with SAMTOOLS v1.9 (RRID:SCR_002105; Li *et al*. 2009). Reads aligned with the soft-masked assembly with rates of 84.5% for the adult male, 87.88% for the adult female, and 91.36% for the caterpillar.

**Fig. 1.**
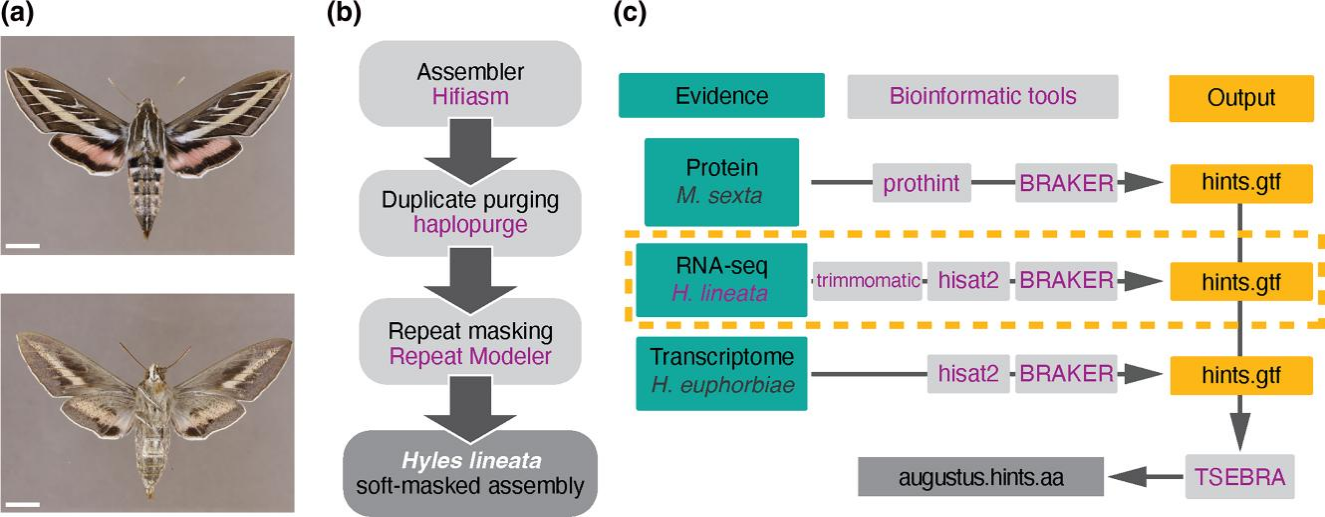
Genome assembly and structural annotation pipeline. a) *Hyles lineata* specimen, dorsal (upper image) and ventral (lower image) from the McGuire Center for Lepidoptera and Biodiversity collection at the Florida Museum of Natural History (MGCL_1032631, CC0 1.0 Public Domain). b) Assembly pipeline. c) Structural annotation pipeline using BRAKER with multiple lines of evidence and the transcript selector, TSEBRA. Scale bar = 1 cm.

We used BRAKER to predict gene models from 3 sources of evidence: amino acid sequences from *M. sexta* (*N* = 53,129) available in the NCBI protein database (BRAKER2; Bruna *et al*. 2020), and 2 separate RNA data sets (BRAKER1; Hoff *et al*. 2016): the transcriptome of the closely related *Hyles euphorbiae* available from the NCBI SRA database (SRR1695429; Barth *et al*. 2018) and RNA-seq reads from 2 life stages and both adult sexes, as described above (Fig. 1). For protein evidence, we first used ProtHint (RRID:SCR_021167) to generate a gff file for the BRAKER annotation. The BRAKER transcript selector, TSEBRA v1.0.3 (Gabriel *et al*. 2021), was employed to unify predictions from these 3 sources (Fig. 1c) and configured such that RNA evidence had greater weight than protein evidence. The resulting gtf file was converted to a fasta file using the perl script, gtf2aa.pl, which is included with the Augustus programming suite. Genome annotations were assessed for completeness using BUSCO with the lepidoptera_odb10 data set (Manni *et al*. 2021). Annotations were further assessed using gFACs, a filtering, analysis, and conversion tool for gene models (Caballero and Wegrzyn 2019).

### Melanin synthesis gene annotation

**Fig. 2.**
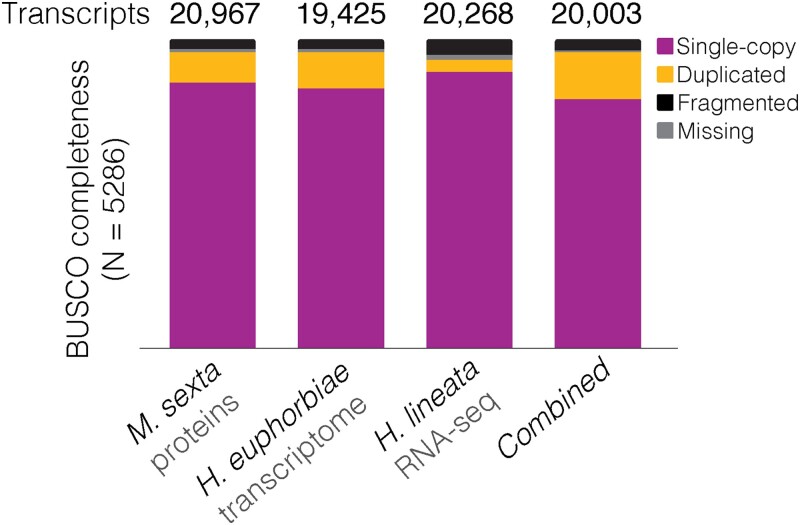
Predicted transcript counts (numbers above bars) from BRAKER annotations using different sources of evidence with BUSCO completeness (color coded). The transcript selector, TSEBRA, was used for the “Combined” category.

Melanin synthesis pathway protein sequences from the domestic silkworm (*Bombyx mori*) available from NCBI (*N* = 11, Supplementary Table 1) were used in BlastP against *H. lineata* amino acid sequences predicted from BRAKER. This was first performed on the TSEBRA unified protein set (Fig. 1c), but we discovered that this annotation likely contained duplicates representing nearly identical transcripts retained from separate lines of evidence, and therefore we used the BRAKER annotation derived from RNA-seq evidence alone (Figs. 1c and 2). Candidate hits were limited to >70% of residues matching the query sequence and an *e*-value <1.0 × 10^−15^. This resulted in a single best match for 7 of the 11 *B. mori* queries. We selected the longest transcript from the remaining hits. To confirm that these curated transcripts were strong candidate melanin pathway gene products, we blasted them against the nonredundant protein database to find sequence similarity with putative homologs from another sphinx moth, the tobacco hornworm (*M. sexta*) and with those of *B. mori*, a relative in the same superfamily, Bombycoidea, and the source of protein sequences used in BlastP. We also included 2 more distantly related moths: the cotton bollworm (*Helicoverpa armigera*) and the striped rice stemborer (*Chilo suppressalis*), and the well-characterized vinegar fly (*Drosophila melanogaster*). The top hit from each species was retained for sequence similarity analysis conducted in R v.4.1.2. To assess whether *H. lineata* sequences share the greatest similarity with *M. sexta*, we first performed multiple sequence alignment with ClustalOmega (msa v.1.24.0, Bodenhofer *et al*. 2015). Distance matrices (function dist.aa) were then used to construct gene trees using the neighbor-joining (function nj) method (Saitou and Nei 1987) in the ape v5.6-2 package. Sequence alignments and gene trees were plotted using ggplot2 v3.4.0 (Wickham 2016), ggmsa v1.3.4 (Zhou *et al*. 2022), and ggtree v.3.2.1 (Guangchuang 2020). Toparslan *et al*. (2020) provided useful guidance for these analyses.

## Results and discussion

### Sequencing and genome assembly

We report a highly contiguous and complete reference genome for the whitelined sphinx (*H. lineata*) assembled from PacBio long-reads. We generated 3.2 million reads with a mean read length of 7,221 bp resulting in a curated assembly of 452 Mb from 52 contigs with an N50 of 14.2 Mb (Table 1). The assembly size is smaller than related sphinx moth genomes (Table 1), but very close to the existing flow cytometry estimate of 449 Mb for the species (Hanrahan and Johnston 2011). However, the k-mer distribution approach performed on the raw reads, which also estimates heterozygosity, suggests a smaller genome size (395 Mb; Supplementary Fig. 1). Notably, other members of the genus *Hyles* have 29 chromosomes (Hundsdoerfer *et al*. preprint), suggesting the *H. lineata* assembly comprised of 52 contigs is very close to being chromosome level. We estimated the repeat content of our curated contigs to be 31.16%, which is lower than reported for *H. euphorbiae* and *Hyles vespertilio* (Table 1).

### Structural annotation

Gene predictions from BRAKER using 3 different sources of evidence resulted in similar numbers of total genes: 20,967 from *M. sexta* protein evidence, 19,425 from the *H. euphorbiae* transcriptome, and 20,268 from *H. lineata* RNA-seq evidence, all with BUSCO completeness scores ≥93% (Fig. 2). Notably, *M. sexta* protein evidence alone results in a predicted gene number and completeness score comparable with those of *H. lineata* RNA-seq evidence. When the 3 separate annotations were merged with the transcript selector, TSEBRA, the resulting gene set (*N* = 20,003) showed a higher number of duplicated genes than separate annotations (15.2%) and only a marginal increase in recovered genes over the RNA-seq evidence (95.9% vs 97.5%, respectively, Fig. 2). Additionally, gFACs-based gene statistics for these BRAKER annotations indicated that the TSEBRA-based models were less complete (Table 2). We therefore proceeded with genes predicted from *H. lineata* RNA-seq evidence for melanin synthesis pathway gene annotation.

**Table 2.** gFACs summary statistics from BRAKER annotations using different sources of evidence.

Annotation evidence	*M. sexta* protein	*H. euphorbiae* transcriptome	*H. lineata* RNA-seq	Combined TSEBRA
Genes	20,971	19,426	20,277	20,053
Monoexonic genes	4,357	3,459	3,423	5,645
Multiexonic genes	16,610	15,966	16,845	14,358
Positive strand genes	10,571	9,762	10,210	10,135
Monoexonic	2,197	1,753	1,732	2,845
Multiexonic	8,370	8,008	8,469	7,240
Negative strand genes	10,400	9,664	10,067	9,918
Monoexonic	2,160	1,706	1,691	2,800
Multiexonic	8,240	7,958	8,376	7,118
Average gene size (bp)	7,217.71	8,420.136	10,948.502	9,545.047
Median gene size (bp)	3,729	4,365	4,794	3,893
Average CDS size (bp)	1,368.45	1,342.304	1,464.59	1,423.496
Median CDS size (bp)	948	882	960	999
Average exon size (bp)	228.482	226.125	214.597	226.545
Median exon size (bp)	155	155	151	154
The following columns do not involve codons				
Complete models	20,225	19,214	19,605	17,296
5′ only incomplete models	453	125	389	2,218
3″ only incomplete models	280	82	261	385
5″ and 3″ incomplete models	9	4	13	104

### Melanin synthesis pathway

**Fig. 3.**
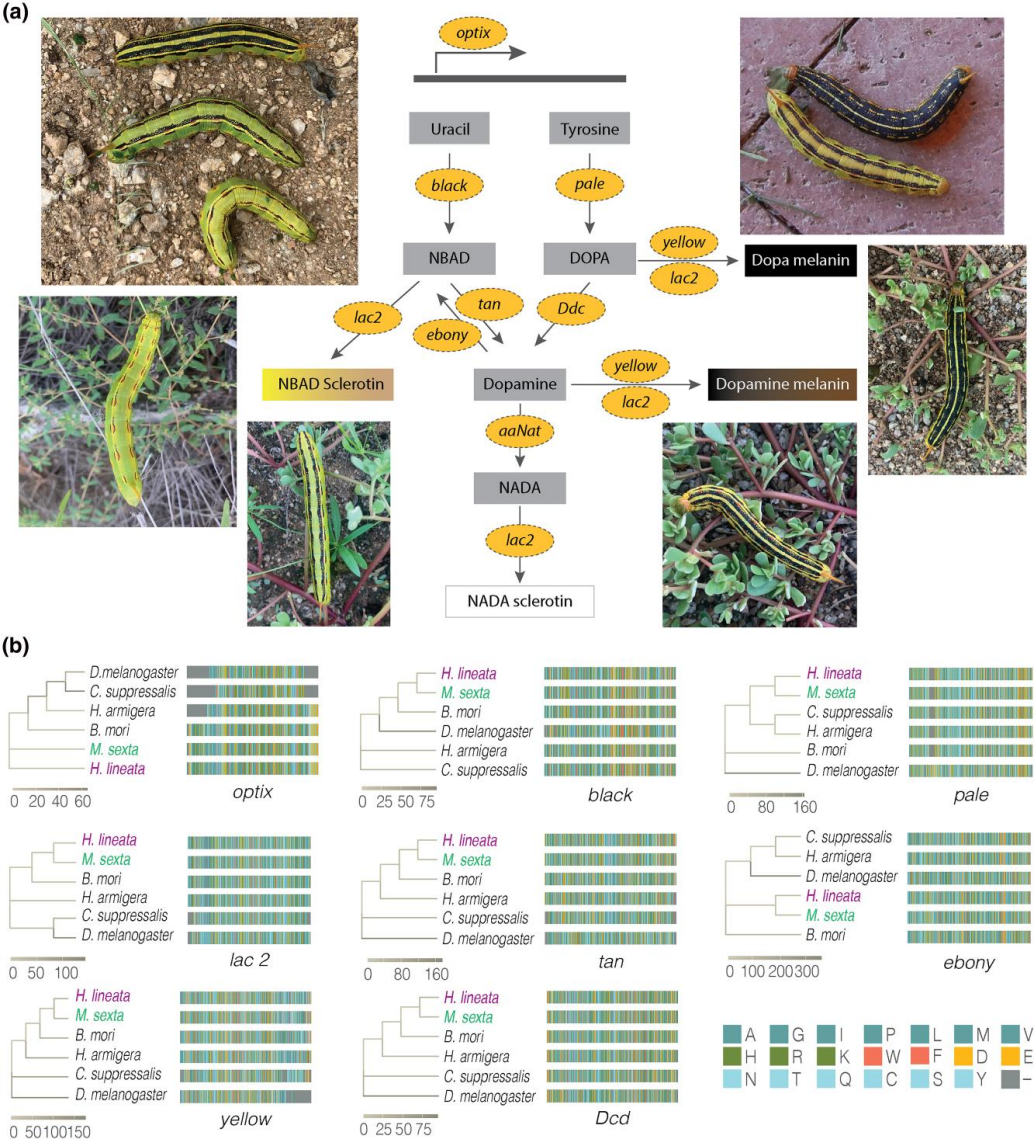
Core melanin synthesis pathway genes annotated from the *Hyles lineata* genome show high conservation with other moths and greatest sequence similarity to the sphinx moth *Manduca sexta*. a) Insect melanin synthesis pathway genes (yellow circles) surrounded by examples of variation in caterpillar cuticular color and pattern. Images provided by Sarah Britton. b) Sequence similarity among amino acids annotated from *H. lineata* and 5 existing insect annotations from the NCBI nonredundant protein database, *M. sexta*, *Bombyx mori*, *Helicoverpa armigera*, *Chilo suppressalis*, and *Drosophila melanogaster*. Branch shade indicates distances between sister branches, with shaded scales for each gene provided below. Amino acid key in lower right: alkyl residues in teal, positively charged residues in green, aromatic residues in coral, negatively charged residues in yellow, and neutral residues in light blue.

The whitelined sphinx has one of the most highly variable larval color polymorphisms among insects. Caterpillars vary in both color and pattern, with cuticular pigmentation ranging from pale yellow to nearly black overlaid with an extensive variety of markings (Fig. 3). Many aspects of this polymorphism are achieved through epigenetic regulation driven by environmental conditions, and *H. lineata* has become an important model system for understanding the evolution and molecular mechanisms of phenotypic plasticity. Here, we leverage a highly conserved component of this system, the core melanin synthesis genes (Fig. 3a), to illustrate the relevance of this genome to a future study of phenotypic plasticity. Predicted protein sequences recovered from the *H. lineata* assembly show high sequence similarity with other moths and the greatest amount of conservation with the closely related sphinx moth, the tobacco hornworm (*M. sexta*, Fig. 3b). Given our ability to confidently curate these genes, along with the phylogenetic position of *H. lineata* as a sister to all other species of *Hyles* (Hundsdoerfer *et al*. 2009), this assembly will bolster existing evolutionary and mechanistic analyses of trait evolution and phenotypic plasticity in sphinx moths.

## Supplementary Material

jkad090_Supplementary_Data

## Data Availability

The PacBio HiFi reads and the genome assembly have been deposited at NCBI under BioProject PRJNA944629 and BioSample accession SAMN33752688. The assembly is named FLMNH_Hlin_v1.0. Output files from blobtools, BRAKER with *H. lineata* RNA-seq data, BUSCO, along with melanin synthesis pathway gene annotation files, are available on DRYAD (https://doi.org/10.5061/dryad.95x69p8q0). Supplemental material available at G3 online.
