# Supplementary material for: A high-quality, long-read genome assembly of the whitelined sphinx moth (Lepidoptera: Sphingidae: Hyles lineata) shows highly conserved melanin synthesis pathway genes
Source: G3 (Bethesda). 2023 Apr 29;13(6):jkad090. doi: 10.1093/g3journal/jkad090 (PMC10234378; doi:10.1093/g3journal/jkad090)
Supplement: jkad090_Supplementary_Data [file jkad090_supplementary_data.pdf]

A high-quality, long-read genome assembly of the whitelined sphinx moth (Lepidoptera: Sphingidae: *Hyles lineata*) shows highly conserved melanin synthesis pathway genes

R. Keating Godfrey[1*], Sarah E. Britton[2], Shova Mishra[3], Jay K. Goldberg[2], Akito Y. Kawahara[1]

Affiliations
[1]McGuire Center for Lepidoptera and Biodiversity, Florida Museum of Natural History, 3215 Hull Rd., University of Florida, Gainesville, FL, 32611
[2]Department of Ecology and Evolutionary Biology, 1041 E. Lowell St., University of Arizona, Tucson, AZ, 85721
[3]Department of Entomology and Nematology,1881 Natural Area Dr., University of Florida, Gainesville FL, 32608

*Corresponding author, rkeating.godfrey@ufl.edu

Supplemental material

Methods

Organism source material

The individual used for sequencing originated from a colony maintained in the Davidowitz Lab at the University of Arizona. This colony was established in 2009, originating from eggs and larvae collected in the Colorado Springs area (Eldorado County, CO). New individuals from the original population are introduced into the lab colony every 4 to 5 years. Lab populations are kept high to maintain diversity (minimum 250 adults per generation). Larvae are raised in the following conditions: 27° C, 40-50% humidity, 16-hour photoperiod, and ad libitum access to an artificial wheat germ-based diet (Davidowitz 2003). Adults are kept in a flight cage where they are provided with ad libitum access to a sponge saturated with a 20% sucrose solution and host plants (*Oenothera caespitosa*) for oviposition. In March of 2022 a male puparium was shipped live from Tucson, AZ to Gainesville, FL and stored in 100% ethanol at -20 °C for two weeks prior to DNA isolation.

DNA Isolation

Thoracic tissue was removed from the puparium and homogenized in lysis buffer and proteinase K. The sample was then nutated for 20 hours at room temperature and incubated at 56 °C for one hour. Hair and scales that would otherwise clog the DNeasy mini spin column membrane were excluded by centrifuging the sample at 20,000 g for 5 minutes and retaining the top liquid portion for all downstream

steps following the standard DNeasy Blood and Tissue Kit protocol. Wide bore pipet tips were used to prevent DNA shearing during transfer steps.

Sequencing

Prior to sequencing, high-integrity and highly pure genomic DNA preparations were evaluated on the Agilent TapeStation, using a genomic tape. Suitable DNA preparations typically had an ABS 260/280 ratio of 1.8-2.0, and an ABS 260/230 ratio of 2.0-2.4. DNA preparations (3-5 μg) were further cleaned using the MoBio PowerClean DNA Cleanup Kit (# 12877-50) kit. Approximately 30% percent of the DNA was lost in this additional clean-up step.

Samples were fragmented down to sizes in the 12-15 kb range using G-tubes in order to optimize the yield of Hi-Fi reads (Covaris Inc. Cat # 520079). Following the fragmentation step, DNA was concentrated using AMPure beads (1:1 bead:sample ratio) and approximately 3 μg of clean DNA was used for the subsequent SMRTbell library construction steps. The library construction steps included: DNA Damage Repair, End Repair/ A-Tailing, SMRT Bell Adaptor ligation, and ExoIII/ExoVII Nuclease treatment. The library construction procedure typically resulted in a functional SMRT bell library (~40% yield from the input amount). The final library (~1.6 μg) was size-selected in the SageELF™ instrument (Cat# ELD 7510), using 0.75% agarose gel cassettes and the 1-18 kb v2 cassette definition program. The desired SageELF fractions were cleaned using AMPure magnetic beads (0.6:1.0 beads to sample ratio) and eluted in 15 ul of 10 nM Tris HCl, pH 8.0. Library fragment size was estimated by the Agilent TapeStation (genomic DNA tapes), and these data were used for calculating molar concentrations. Typically, ELF fractions in wells 4-7 contained library fragments of the desired length (10-15 k). The on-plate loading concentration was 75 pM. Other run parameters included: diffusion loading, 2-hr pre-extension, on-instrument Hi-Fi reads generation and 30 hr movies. The instrument used Sequencing Kit 2.0 (PacBio Cat. # 101-389-001) and Instrument Control SW Version 11.0 (SMRT Link 11.0).

Assembly and analysis

Three low-coverage contigs were identified as *Firmicutes*: ptg000100l, and ptg000179l, ptg000214l. We also identified contig ptg000097l as mitochondrial DNA based on sequence similarity with other insect mitochondrial genes.

References

Davidowitz, G., D'Amico, L.J., Nijhout, H.F., 2003. Critical weight in the development of insect body size. Evol Dev. 5(2): 188–197

Table S1. Subset of core melanin synthesis pathway genes (Sugumaran & Barek 2016) from *Bombyx mori* used to identify putative homologs in *Hyles lineata* annotation.

Gene	Protein	Accession number	Putative homolog	Percent identical positions
aaNAT	N-acetyl transferase	NP_001073122.1	g8895.t1	80.519
black	Aspartate decarboxylase	NP_001296491.1	g17810.t1	86.068
Dat	Dopamine transporter	NP_001037362.2	g205.t1	92.336
Ddc	Dopa decarboxylase	AAK48988.1	g3482.t1	89.286
ebony	NBAD synthetase	NP_001138793.1	g14996.t1	78.748
GTPCHI	GTP cyclohydrolase	NP_001138797.1	g5875.t1	97.27
lac2	Laccase2	XP_037869032.1	g264.t1	86.728
optix	Optix	XP_004930699.2	g14033.t1	96.35
tan	NBAD hydrolase	NP_001170882.1	g2242.t1	72.589
TH/pale	tyrosine hydroxylase	NP_001138794.1	g2304.t1	95.187
yellow	DCDT, DPT, others	ABC96700.2	g4897.t1	74.088

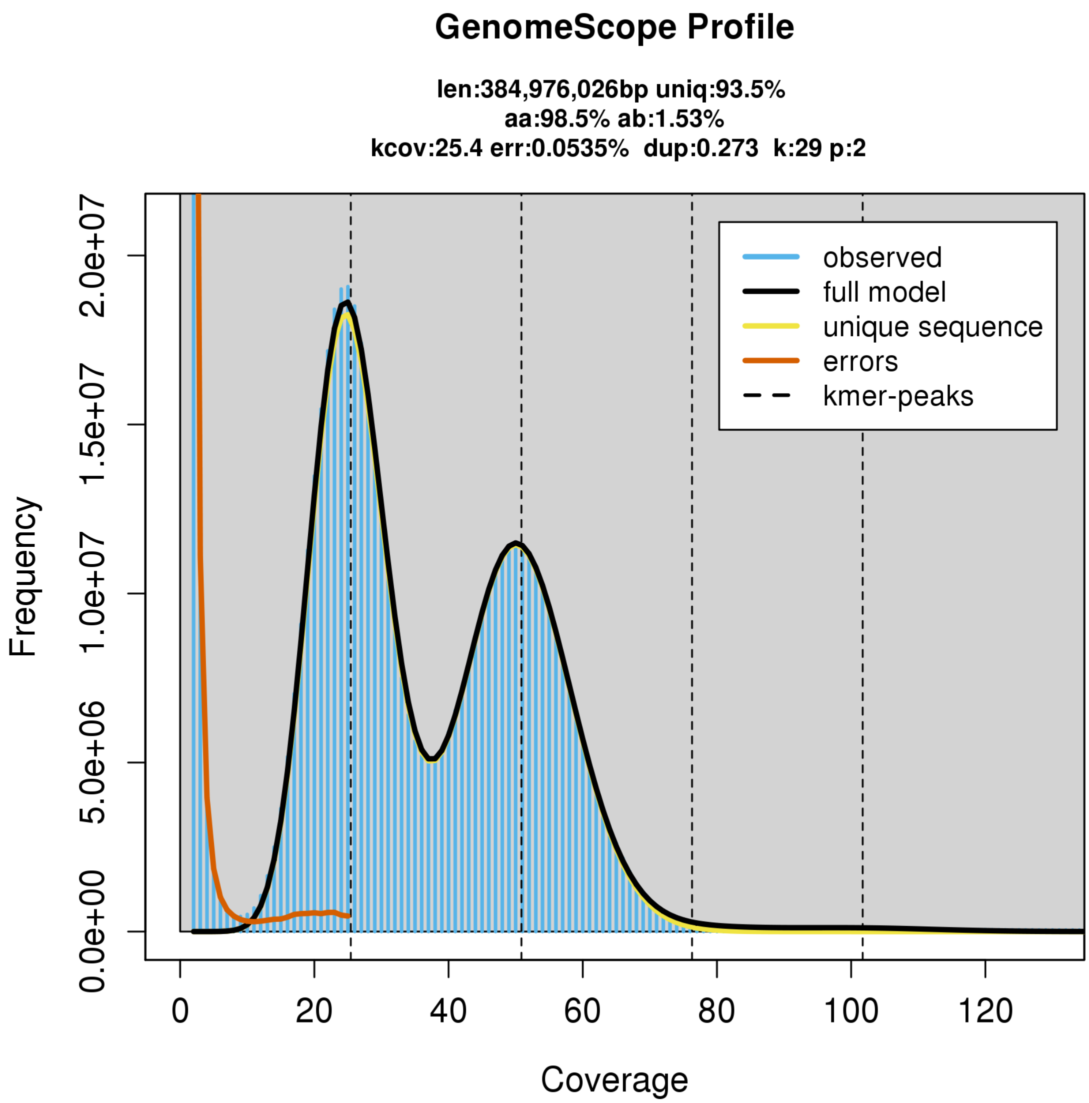

Figure S1. Genome size and heterozygosity assessed from raw read data using K-Mer counter and plotted using GenomeScope 2.0.

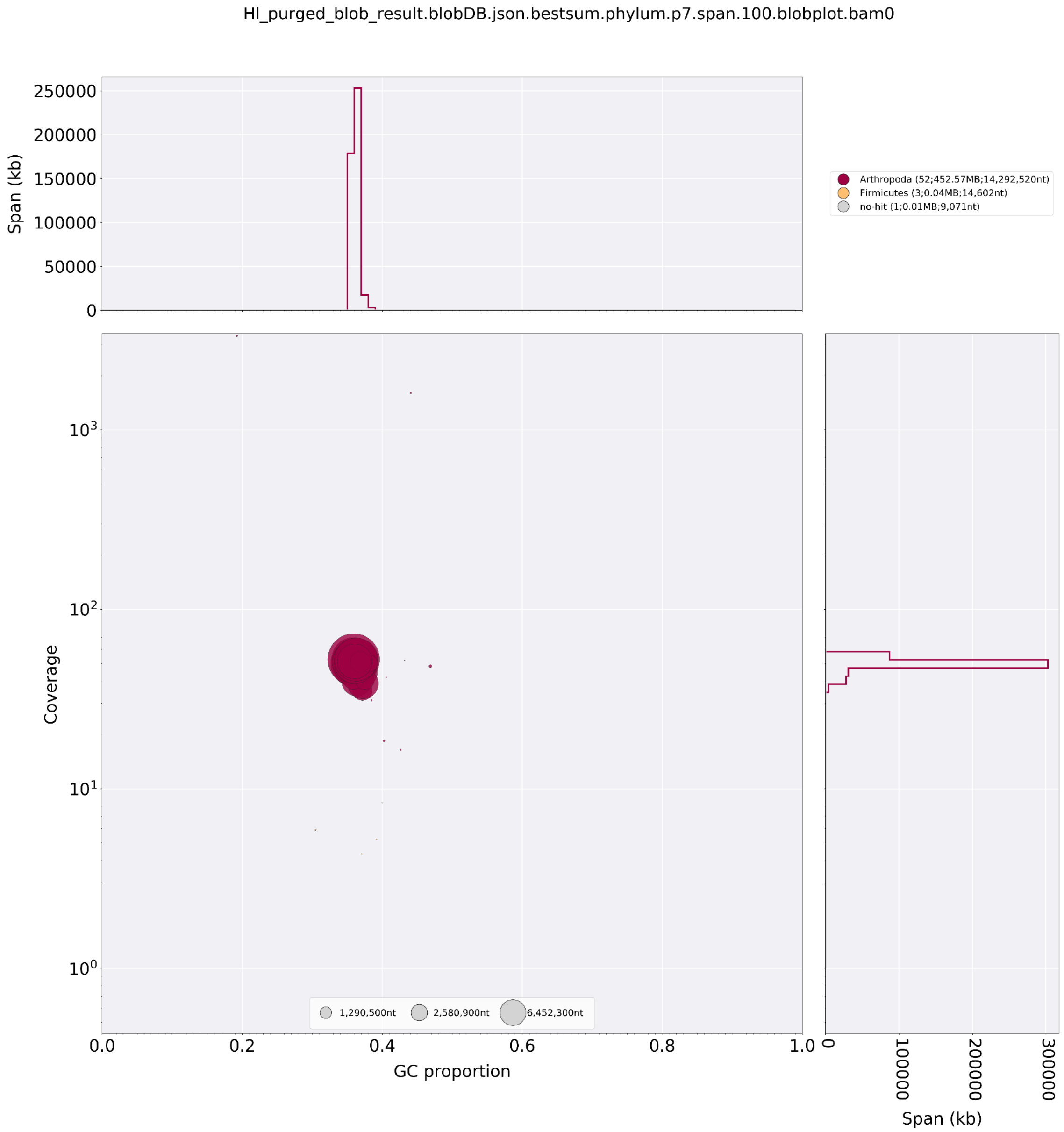

Figure S2. Contamination check displayed using Blopblot 1.0.
